# IL-36β Promotes CD8^+^ T Cell Activation and Antitumor Immune Responses by Activating mTORC1

**DOI:** 10.3389/fimmu.2019.01803

**Published:** 2019-08-07

**Authors:** Xin Zhao, Xiaojuan Chen, Xinghua Shen, Peijun Tang, Chen Chen, Qitai Zhu, Muyao Li, Rui Xia, Xi Yang, Chao Feng, Xinguo Zhu, Yibei Zhu, Zhongwen Sun, Xueguang Zhang, Binfeng Lu, Xuefeng Wang

**Affiliations:** ^1^Department of General Surgery, The First Affiliated Hospital, Soochow University, Suzhou, China; ^2^Department of Biochemistry and Molecular Biology, School of Biology and Basic Medical Sciences, Soochow University, Suzhou, China; ^3^Jiangsu Institute of Clinical Immunology, The First Affiliated Hospital of Soochow University, Suzhou, China; ^4^Jiangsu Key Laboratory of Clinical Immunology, Soochow University, Suzhou, China; ^5^Jiangsu Key Laboratory of Gastrointestinal Tumor Immunology, The First Affiliated Hospital of Soochow University, Suzhou, China; ^6^Department of Gastroenterology, The First Affiliated Hospital of Soochow University, Suzhou, China; ^7^Department of Pulmonary Tuberculosis, The Affiliated Hospital for Infectious Diseases of Soochow University, Suzhou, China; ^8^Department of Oncology, The Second Affiliated Hospital of Soochow University, Suzhou, China; ^9^School of Medicine, Tsinghua University, Peking, China; ^10^Institute of Translational Medicine, Soochow University, Suzhou, China; ^11^Department of Immunology, School of Biology and Basic Medical Sciences, Soochow University, Suzhou, China; ^12^Institute of Medical Biotechnology, Suzhou Vocational Health College, Vocational Health College, Suzhou, China; ^13^Department of Immunology, University of Pittsburgh School of Medicine, Pittsburgh, PA, United States

**Keywords:** IL-36β, CD8^+^ T cells, mTORC1, antitumor immune responses, tumor microenvironment

## Abstract

Cytokine-amplified functional CD8^+^ T cells ensure effective eradication of tumors. Interleukin 36α (IL-36α), IL-36β, and IL-36γ share the same receptor complex, composed of the IL-36 receptor (IL-36R), and IL-1RAcP. Recently, we revealed that IL-36γ greatly promoted CD8^+^ T cell activation, contributing to antitumor immune responses. However, the underlying mechanism of IL-36-mediated CD8^+^ T cell activation remains understood. In the current study, we proved that IL-36β had the same effect on CD8^+^ T cell as IL-36γ, and uncovered that IL-36β significantly activated mammalian target of rapamycin complex 1 (mTORC1) of CD8^+^ T cells. When mTORC1 was inhibited by rapamycin, IL-36β-stimulated CD8^+^ T cell activation and expansion was drastically downregulated. Further, we elucidated that IL-36β-mediated mTORC1 activation was dependent on the pathway of phosphatidylinositol 3 kinase (PI3K)/Akt, IκB kinase (IKK) and myeloid differentiation factor 88 (MyD88). Inhibition of PI3K or IKK by inhibitor, or deficiency of MyD88, respectively, suppressed mTORC1 signal, causing arrest of CD8^+^ T cell activation. Additionally, it was validated that IL-36β significantly promoted mTORC1 activation and antitumor function of CD8^+^ tumor-infiltrating lymphocytes (TILs) *in vivo*, resulting in inhibition of tumor growth and prolongation of survival of tumor-bearing mice. Taken together, we substantiated that IL-36β could promote CD8^+^ T cell activation through activating mTORC1 dependent on PI3K/Akt, IKK and MyD88 pathways, leading to enhancement of antitumor immune responses, which laid the foundations for applying IL-36β into tumor immunotherapy.

## Introduction

CD8^+^ T cells exert a predominant effect in eradicating tumors (1, 2). However, owing to inhibition by immune checkpoints, regulatory T cells (Tregs), and myeloid-derived suppressor cells (MDSCs) (3–5), CD8^+^ TILs are functionally suppressed (6). Thus, restoring antitumor function of CD8^+^ TILs is considered as an effective method to eliminate tumors. Indeed, current immunotherapy, based on blockade of checkpoint molecules such as programmed death-1 (PD-1) and cytotoxic T lymphocyte-associated antigen-4 (CTLA-4) is achieving great clinical success (7, 8). However, such therapeutic regimens mainly depend on the levels of existing tumor-specific TILs (9). Thus, a considerable proportion of tumor patients have no responses to treatment with PD-1, PD-1 ligand-1 (PD-L1), or CTLA-4 antibody (10, 11). Therefore, it remains to explore more effective means to boost CD8^+^ TILs and break down immune tolerance.

Fundamentally, generation of effector CD8^+^ T cells are established on the basis of energy and nutrient-required expansion. Hence, those signal pathways mediating CD8^+^ T cell anabolism are extremely important for exertion of CD8^+^ T cell function (12). Recently, mammalian target of rapamycin (mTOR) is emerging as a central integrator of diverse signals and controls multiple metabolic programs (13). mTOR forms two functionally distinct signaling complexes, mTOR complex 1 (mTORC1) and mTOR complex 2 (mTORC2). mTORC1, composed of mTOR, Raptor and mLST8, can be inhibited by rapamycin, while mTORC2, composed of mTOR, Rictor, Sin1 and mLST8, is insensitive to rapamycin (14). Activation of mTORC1 phosphorylates eukaryotic initiation factor 4E-binding protein 1 (4EBP1) and S6 kinase (S6K1 or p70S6K), then phosphorylates the ribosomal protein S6, and then results in cellular biosynthesis. It has been well-documented that engagement of cytokine receptors, antigen receptors, or Toll-like receptors (TLRs) triggers mTORC1 activation, leading to T cell anabolism, and expansion (15, 16).

As the newly discovered IL-1 family members, IL-36α, IL-36-β, and IL-36γ interact with the same receptor complex, composed of IL-36R (also known as IL-1Rrp2 or IL-1RL2) and interleukin-1 receptor accessory protein (IL-1RAcP) (17). IL-36R is expressed on monocytes, dendritic cells (DCs) and CD4^+^ T cells. IL-36 cytokines exert potent stimulatory effect on these immune subsets (18, 19). Recently, we have demonstrated that IL-36R is also expressed by CD8^+^ T cells (20, 21). IL-36/IL-36R signal plays a remarkable role in promoting CD8^+^ T cell activation, proliferation and cytokine production, and enhances CD8^+^ T cell-mediated antitumor immune responses (21). Therefore, IL-36 cytokines can be considered as potential materials to provoke CD8^+^ TIL antitumor function. However, the underlying signal mechanism is still not explored. It remains to be figured out how IL-36 cytokines activate CD8^+^ T cells.

Therefore, to explore whether and how IL-36 cytokine promoting CD8^+^ T cell activation by mTORC1 pathway will deepen our understanding for the role and mechanism of IL-36/IL-36R signal on antitumor function. In this paper, we substantially demonstrated that IL-36β could greatly promote CD8^+^ T cell activation and expansion through activating mTORC1 dependent on PI3K/Akt, IKK, and MyD88 pathways, and tumoral expression of IL-36β could significantly enhance mTORC1 activation and antitumor function of CD8^+^ TILs.

## Results

### IL-36β Promoted CD8^+^ T Cell Activation, Expansion, and Cytokine Production

**Figure 1:**
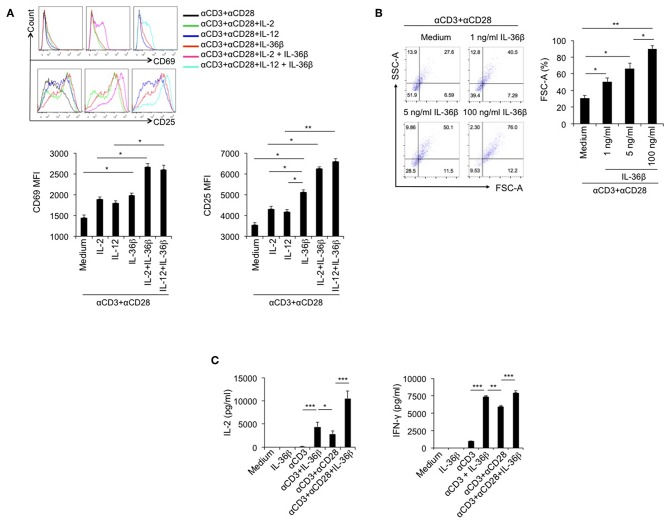
IL-36β promoted naïve CD8^+^ T cell activation, expansion and IL-2 and IFN-γ production. Naïve CD8^+^ T cells were isolated from C57BL/6j mice and stimulated with or without plate-bound 10 μg/ml anti-CD3 mAb, or in combination with 5 μg/ml anti-CD28 mAb, in the presence or absence of IL-36β (100 ng/ml), human IL-2 (20 U/ml), IL-12 (10 ng/ml) alone, or in combination for various lengths of time. **(A)** Naïve CD8^+^ T cells were stimulated with anti-CD3 and anti-CD28 mAbs in the presence of cytokines as indicated and the expression of CD25 and CD69 on the surface of at 24 h was measured by flow cytometry. **(B)** Naïve CD8^+^ T cells were stimulated with anti-CD3 and anti-CD28 mAbs in the presence or absence of IL-36β and cell sizes (forward scatter) at 72 h were determined by flow cytometry. **(C)** Naïve CD8^+^ T cells were stimulated as indicated. The levels of IL-2 and IFN-γ at 6 h were measured by ELISA method. Data are shown as mean ± SEM. *p < 0.05, **p < 0.01, and ***p < 0.001 by one way ANOVA test. Data are shown from one of three independent experiments with similar results.

Since IL-36R is expressed on CD8^+^ T cells and IL-36γ can activate CD8^+^ T cells (21), we speculated that the other two IL-36 cytokines had the same function. Here, we investigated the stimulatory effect of IL-36β on CD8^+^ T cells. Naïve CD8^+^ T cells were isolated from C57BL/6j mice and stimulated with or without plate-bound anti-CD3 mAb, or in combination with plate-bound anti-CD28 mAb, in the absence or presence of IL-36β, IL-2, and IL-12 alone or in combination. As expected, upon stimulation for 24 h, both IL-2 and IL-12 enhanced the expression of CD69 and CD25, two markers of T cell activation. Interestingly, IL-36β obviously upregulated CD69 and CD25 and was more effective in increasing CD25 expression than IL-2 and IL-12 (Figure 1A). Surprisingly, we found that IL-36β, when combined with IL-2 or IL-12, respectively, further increased CD69 and CD25 expression (Figure 1A). Thereby, IL-36β could profoundly promote the expression of activation markers CD25 and CD69 on naïve CD8^+^ T cells at the early stage and has a synergistic effect with IL-2 and IL-12, respectively. Furthermore, we analyzed the effect of IL-36β on expanding CD8^+^ T cell since cell size is the material basis for exertion of T cell function. We noted that IL-36β could evidently enlarge CD8^+^ T cells in a dose-dependent manner (Figure 1B), implying that IL-36β increased the biomass production during T cell activation. Previously IL-36γ was reported to promote IL-2 and IFN-γ production upon stimulation for 24 h (21). Interestingly, in the current study we demonstrated that even at 6 h, IL-36β could greatly promote the production of both IL-2 and IFN-γ in combination with anti-CD3 mAb, whether in the presence of anti-CD28 antibody or not (Figure 1C). Additionally, the kinetic analysis at mRNA and protein levels demonstrated that IL-36β could persistently upregulate IFN-γ and IL-2 at 24, 48, and 72 h (Figures S1A,B). At the same time, the effect of IL-36β upregulating IFN-γ was further substantiated by determination through FACS (Figure S1C). Moreover, the cytotoxic molecules Granzyme B and C were also increased in CD8^+^ T cells upon IL-36β stimulation (Figure S1A). Taken together, these findings indicated that IL-36/IL-36R signal exerted a fantastic stimulatory effect on CD8^+^ T cells.

### IL-36β-Promoted CD8^+^ T Cell Activation Was Dependent on mTORC1

**Figure 2:**
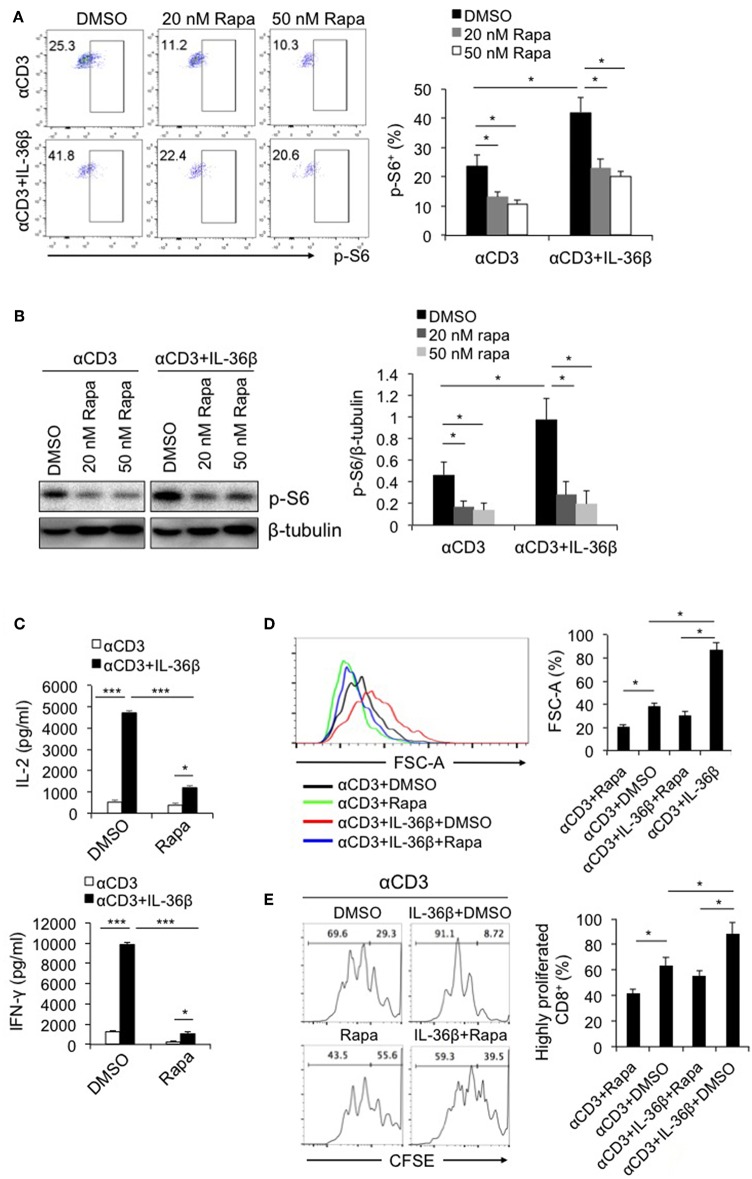
IL-36β-mediated CD8^+^ T cell activation was dependent on mTORC1. Naïve CD8^+^ T cells were isolated from C57BL/6j mice and stimulated with plate-bound 10 μg/ml anti-CD3 mAb, in the presence or absence of IL-36β (100 ng/ml), or rapamycin (20 or 50 nM) for various lengths of time. **(A)** Phosphorylation of ribosomal protein (p-S6) was measured by flow cytometry at 48 h. **(B)** Phosphorylation of ribosomal protein (p-S6) was measured by western blot at 48 h. **(C)** The levels of IL-2 and IFN-γ production in the supernatants were measured by ELISA method at 48 h. **(D)** Cell sizes (forward scatter) at 72 h were determined by flow cytometry. **(E)** Cell proliferation based on CFSE dilution assay at 72 h were determined by flow cytometry. Data are shown as mean ± SEM. *p < 0.05, **p < 0.01 and ***p < 0.001 by unpaired t test. The experiment was repeated independently three times.

Since IL-36 cytokines can greatly promote CD8^+^ T cell activation, we intend to explore the underlying mechanism. As described previously, mTORC1 plays a central role in promoting activation and biomass synthesis of T cells through integrating diverse signals (22). Consequently, we sought to investigate whether IL-36β-mediated CD8^+^ T cell activation is dependent on mTORC1. Naïve CD8^+^ T cells were stimulated with plate-bound anti-CD3 and anti-CD28 mAbs in the presence or absence of IL-36β or rapamycin. Upon stimulation for 48 h, the level of phosphorylated ribosomal protein S6 (p-S6) was determined by flow cytometry and western blot, respectively. Interestingly, p-S6 level was significantly increased by IL-36β, while rapamycin greatly inhibited IL-36β-mediated upregulation of p-S6 (Figures 2A,B). Further, we examined the influence of inhibition of mTORC1 signal on IL-36β-boosted CD8^+^ T cell activation. IL-36β could remarkably enhance the levels of both IL-2 and IFN-γ production in a dose-dependent manner (Figure 2C and Figure S2A). However, in the presence of rapamycin, IL-36β-mediated upregulation of IL-2, and IFN-γ was greatly suppressed (Figure 2C and Figure S2A). At the same time, IL-36β profoundly enlarged CD8^+^ T cell size in a dose-dependent manner, but rapamycin significantly inhibited this effect (Figure 2D and Figure S2B). In addition, we inspected the importance of mTORC1 signal on IL-36β-driven CD8^+^ T cell proliferation. Naïve CD8^+^ T cells were stained with carboxyfluorescein succinimidyl ester (CFSE) and then stimulated with anti-CD3 mAb in the presence or absence of IL-36β or rapamycin. Upon stimulation for 72 h, the proliferation of CD8^+^ T cells was quantified by analyzing the CFSE dilution by flow cytometry. Compared with the control, CD8^+^ T cells cultured in the presence of IL-36β proliferated at much higher levels in a dose-dependent manner (Figure 2E and Figure S2B). However, in the presence of rapamycin, CD8^+^ T cell proliferation mediated by IL-36β was obviously inhibited (Figure 2E and Figure S2B). Additionally, we investigated the expression levels of p-S6 in functional CD8^+^ T cells characterized by IFN-γ production. The results showed that ~90% of IFN-γ^+^ CD8^+^ T cells presented p-S6 positive both in the group with IL-36β stimulation and the control group (Figure S2C). Thereby, these data indicated that IL-36β could significantly boost mTORC1 signal of CD8^+^ T cells and IL-36β-mediated CD8^+^ T cell activation was dependent on mTORC1. However, it was yet unknown which signal pathways were involved in IL-36β- triggered mTORC1 activation.

### PI3K/Akt and IKK Pathways Were Involved in IL-36β-Mediated mTORC1 Activation of CD8^+^ T Cells

**Figure 3:**
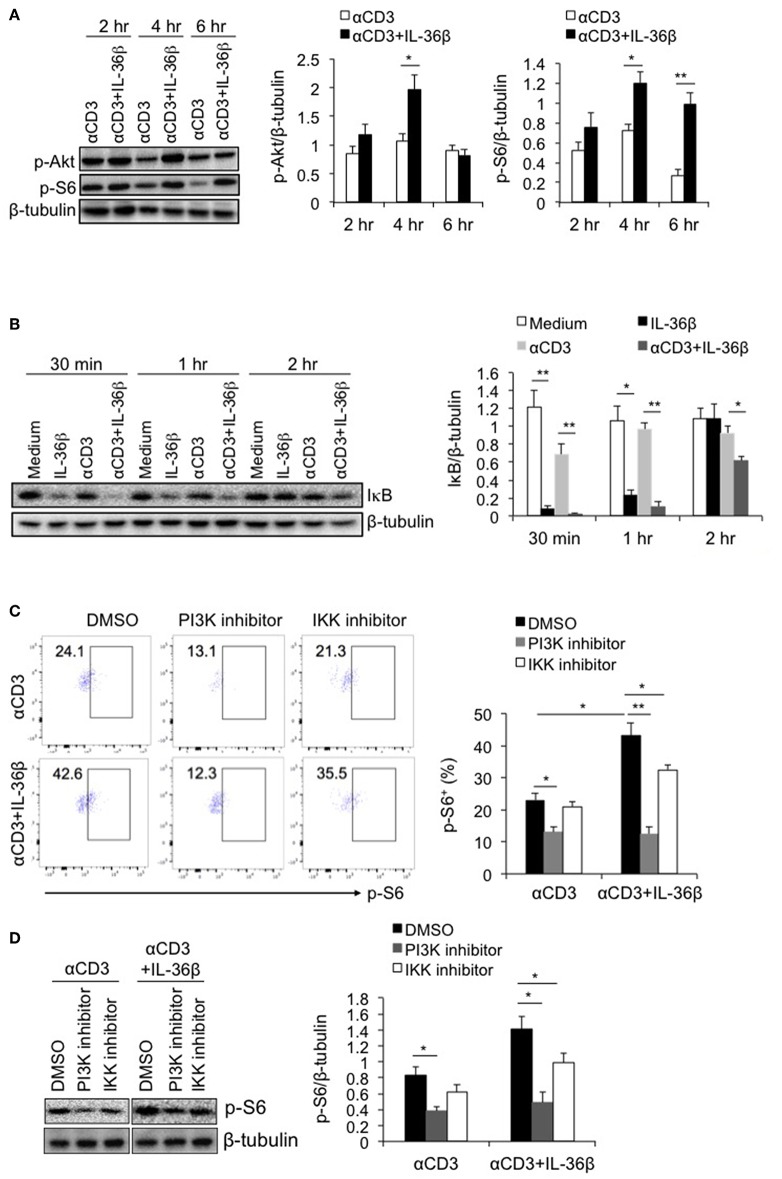
PI3K/Akt and IKK pathways were involved in IL-36β-mediated mTORC1 activation of CD8^+^ T cells. Naïve CD8^+^ T cells were isolated from C57BL/6j mice and stimulated with or without plate-bound 10 μg/ml anti-CD3 mAb, in the presence or absence of IL-36β (100 ng/ml) for various lengths of time. **(A)** Phosphorylation of Akt and S6 at different time points as indicated were determined by western blot. **(B)** Degradation of IκB at different time points as indicated was measured by western blot. **(C,D)** In the presence or absence of PI3K or IKK inhibitor, phosphorylation of S6 at 48 h was measured by flow cytometry **(C)** and western blot **(D)**. Data are shown as mean ± SEM. *p < 0.05 and **p < 0.01 by unpaired *t-*test. The experiment was repeated independently three times.

Multiple signals including costimulatory molecules, cytokines and cell stress can activate mTORC1 through diverse intracellular pathways, leading to T cell activation (13, 23). Among those intracellular pathways, PI3K/Akt has been well documented to drive mTORC1 activation (22). PI3K, after being recruited to cellular member, stimulates the production of phosphatidylinositol (3,4,5)-trisphosphate (PIP3), resulting in phosphorylating serine-threonine protein kinase Akt. Then activated Akt phosphorylates the tuberous sclerosis complex 2 (TSC2), causing mTORC1 activation (24). Since IL-36β significantly promoted mTORC1 activation, we postulated that such an effect was probably dependent on PI3K/Akt pathway. Thus, we first evaluated the role of IL-36β in activating PI3K/Akt pathway. Naïve CD8^+^ T cells were stimulated with plate-bound anti-CD3 mAb in the presence or absence of IL-36β. The phosphorylation of Akt (p-Akt) was determined by western blot. The results showed that compared with the control, the level of p-Akt was obviously enhanced by IL-36β at 4 h (Figure 3A). Intriguingly, the level of p-S6 was subsequently upregulated (Figure 3A).

Moreover, inhibitors of nuclear factor kappa-B kinases (IKKs), including IKKα and IKKβ, have been recently reported to have a stimulatory function on mTORC1 activation (25, 26). mTORC1 can be activated by IKKα through phosphorylating mTOR at serine 1415, and by IKKβ through suppressing TSC1 (25, 26). Therefrom, we examined whether induction of IKK activity was involved in IL-36β-mediating mTORC1 activation of CD8^+^ T cells. Naïve CD8^+^ T cells were stimulated with or without plate-bound anti-CD3 mAb, in the presence or absence of IL-36β. Then IKK activity was analyzed through determining IκB level by western blot, since IKKs phosphorylate IκB, causing its degradation. The results showed that upon stimulation for 30 min and 1 h, IL-36β alone, obviously accelerated the degradation of IκB, presenting more significantly effect than TCR signal in our experiment. When in combination with TCR signal, IL-36β greatly promoted IκB further degradation, the band of which was almost invisible at 30 min (Figure 3B). These data indicated that IL-36β could profoundly increase IKK activity.

Then, we investigated whether IL-36β-stimulated PI3K/Akt and IKK pathways were involved in mTORC1 activation. The inhibitor of PI3K or IKK, or the control dimethyl sulfoxide (DMSO), was respectively, added to the culture of naïve CD8^+^ T cells, which were stimulated with plate-bound anti-CD3 mAb, in the presence or absence of IL-36β. The levels of p-S6 were measured by flow cytometry and western blot. It was found that when PI3K/Akt pathway was blocked, TCR signal-triggered phosphorylation of S6 protein was decreased. At the same time, IL-36β-mediated S6 phosphorylation was significantly suppressed, the level of which was downregulated approximately equal to that of CD8^+^ T cells without IL-36β stimulation in the presence of PI3K inhibitor (Figures 3C,D). Thereby, these data indicated that PI3K/Akt pathway had a central role in IL-36β-mediated mTORC1 activation. Moreover, we found that when IKK was inhibited, the phosphorylation of S6 triggered by IL-36β was partially inhibited (Figures 3C,D), suggesting that IKK pathway was also involved in IL-36β promoted mTORC1 activation.

### MyD88 Deficiency Inhibited IL-36β-Mediated mTORC1 Activation of CD8^+^ T Cells and Resulted in Suppression of CD8^+^ T Function

**Figure 4:**
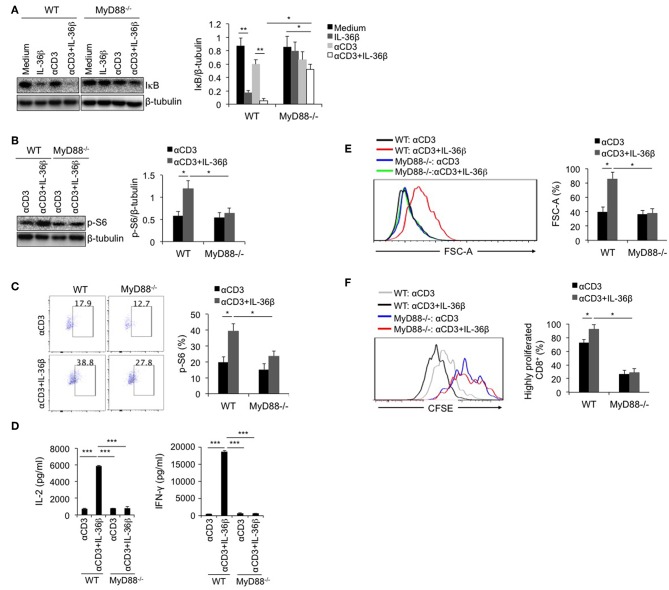
MyD88 deficiency inhibited IL-36β-mediated mTORC1 activation of CD8^+^ T cells and resulted in suppression of CD8^+^ T function. Naïve CD8^+^ T cells were, respectively, isolated from C57BL/6j and MyD88^−/−^ mice and stimulated with or without plate-bound 10 μg/ml anti-CD3 mAb, in the presence or absence of IL-36β (100 ng/ml) for various lengths of time. **(A)** Degradation of IκB at 1h upon stimulation was measured by western blot. **(B)** Phosphorylation of ribosomal protein S6 at 48 h was measured and then analyzed by western blot. **(C)** Phosphorylation of ribosomal protein S6 at 48 h was measured and then analyzed by flow cytometry. **(D)** The levels of IL-2 and IFN-γ production in the supernatants were measured by ELISA method at 48 h. ***p < 0.001 by unpaired *t-*test. **(E)** Cell sizes (forward scatter) at 72 h were determined by flow cytometry. **(F)** Cell proliferation based on CFSE dilution assay at 72 h were determined by flow cytometry. Data are shown as mean ± SEM. *p < 0.05, **p < 0.01, and ***p < 0.001 by unpaired *t-*test. The experiment was repeated independently three times.

As the shared co-receptor of IL-1, IL-33, and IL-36 cytokines, IL-1RAcP associates with IL-1R, ST2, and IL-36R, and induces MyD88-dependent signal (27). Thereby, as a key adaptor, MyD88 plays an important role in transducing these cytokine-mediated signals. At the same time, MyD88 is an important upstream signal leading to IKK/NF-κB pathway. Here, we postulated that IL-36β-mediated mTORC1 activation was closely associated with MyD88. Naïve CD8^+^T cells were, respectively, isolated from WT C57BL/6j and MyD88^−/−^ mice, stimulated with or without anti-CD3 mAb in the presence or absence of IL-36β, and then the levels of IκB, and p-S6 were measured by western blot and (or) flow cytometry. We found that in MyD88^−/−^ CD8^+^ T cells, IκB degradation mediated by IL-36β in combination with anti-CD3 mAb was significantly restored (Figure 4A). In addition, we noted that in combination with TCR signal stimulation, IL-36β obviously increased p-S6 level in WT CD8^+^ T cells upon stimulation for 24 h (Figures 4B,C). Whereas, such an effect was inhibited in MyD88^−/−^ CD8^+^ T cells (Figures 4B,C). Therefore, as a key adaptor, MyD88 had a critical role in IL-36β-mediated mTORC1activation. Further, we examined the influence of MyD88 deficiency on mTORC1-associated CD8^+^ T cell function. Upon stimulation for 48 h, IL-36β-promoted IL-2, and IFN-γ production was drastically inhibited in MyD88^−/−^ CD8^+^ T cells (Figure 4D). We also noted that CD8^+^ T cell size were enlarged by IL-36β upon stimulation for 72 h whereas MyD88 deficiency suppressed this effect (Figure 4E). In addition, IL-36β-mediated CD8^+^ T cell proliferation, which was quantified through analyzing the CFSE dilution by flow cytometry, was also reduced in MyD88^−/−^ CD8^+^ T cells (Figure 4F). These data indicated that MyD88 was indispensible for IL-36β-mediated CD8^+^ T cell expansion, proliferation and cytokine production through activating mTORC1.

### IL-36β Promoted mTORC1 Activation and Antitumor Function of CD8^+^ TILs

Since IL-36β could promote CD8^+^ T cell activation by stimulating mTORC1 *in vitro*, we wondered whether IL-36β has a similar function on CD8^+^ TILs in the tumor microenvironment *in vivo*.

First, B16 melanoma cells were used to generate control B16-vector (B-16-vec) and B16-IL-36β cell clones that expressed mouse IL-36β. The expression of IL-36β in B16 cell lines was detected by QRT-PCR method (Figure S3A). B16-vec and B16-IL-36β were evidenced to present similar growing capacities in culture (Figure S3B). At the same time, IL-36R expression in B16 cell lines were examined. The results showed that IL-36R presented a very low expression level on B16-vec and B16-IL-36β cells compared with CD4^+^ and CD8^+^ T cells and splenocytes (Figure S3C). In addition, in order to analyzed whether IL-36β has any cell-intrinsic effect on tumor cell growth, B16-vec and B16-IL-36β cells were, respectively, injected into nude mice intradermally. Tumor growth was monitored every 2 days. It was found that B16-IL-36β grew slightly faster than B16-vec at the later stage of tumor growth (Figure S3D).

**Figure 5:**
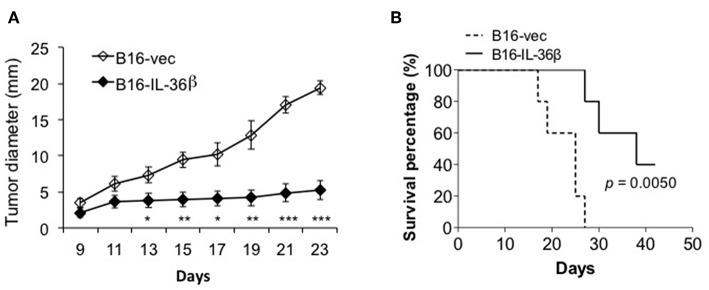
The expression of IL-36β in tumor microenvironment suppressed tumor progression and prolonged the survival of tumor-bearing mice. **(A)** 1 × 10^5^ B16-vector (B16-vec) or B16-IL-36β cells were injected intradermally into C57BL mice (*n* = 5) and size of the tumor was monitored every 2 days. Data are shown as mean ± SEM. Comparison was performed between B16-vec and B16-IL-36 β. *p < 0.05, **p < 0.01 and ***p < 0.001 by unpaired *t* test. **(B)** 1 × 10^5^ B16-vector (B16-vec) or B16-IL-36β cells were injected intradermally into B6 mice. Survival of mice was monitored. Five mice were in each group. *p*-value was calculated by Log-rank Test. The experiment was repeated independently three times.

Then, B16-vec and B16-IL-36β cells were injected into C57/BL6 mice intradermally. Tumor growth was monitored every 2 days. As expected, tumor growth was obviously inhibited upon IL-36β expression (Figure 5A). At the same time, the expression of IL-36β in B16 cells significantly extended the survival of tumor-bearing mice (Figure 5B). In addition, intraperitoneal injection of recombinant IL-36β protein could also inhibit tumor growth, but exerted a relatively lower effect compared with tumor-expressed IL-36β (Figure S4A). This may be due to the fact that IL-36β in the tumor microenvironment locally is more effective than systemic administration. Moreover, for investigating whether rapamycin can restore B16 tumor growth by inhibiting IL-36β-promoted CD8^+^ T function, rapamycin was injected at day 9 after tumor cell injection. Interestingly, upon rapamycin injection, B16-vec tumor growth was partially inhibited at the late stage of tumor growth. Upon rapamycin injection, B16-IL-36β tumor growth could be partially restored but later the tumors keep similar size to B16-IL-36β tumor without rapamycin injection (Figure S4B). This could be explained that rapamycin not only suppressed the function of IL-36β-activated CD8^+^ T cells, but also inhibited the growth of B16 tumors.

**Figure 6:**
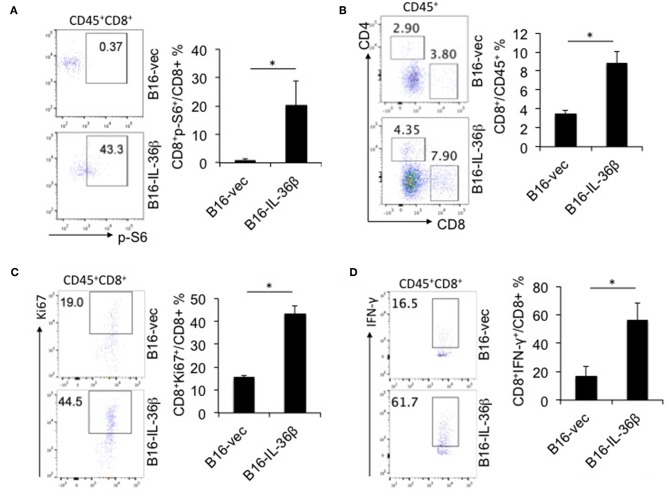
IL-36β promoted mTORC1 activation and antitumor function of CD8^+^ TILs. 1 × 10^5^ B16-vector (B16-vec) or B16-IL-36β cells were injected intradermally into B6 mice (*n* = 5). On day 23, tumors were resected and processed to generate a single cell suspension. **(A)** Representative flow cytometric plots and analyzed results showed p-S6 level of CD8^+^ TILs. **(B)** Representative flow cytometric plots and analyzed results showed the percentages of CD8^+^ TILs within the gated CD45^+^ population in tumors. **(C)** Representative flow plots and analyzed results showed the percentages Ki67^+^ cells within CD45^+^CD8^+^TILs. **(D)** Representative flow plots and analyzed results showed the percentages IFN-γ^+^ cells within CD45^+^CD8^+^ TILs. Data (mean ± SEM) are averages of five samples. *p < 0.05, analyzed by two-tailed unpaired Student's *t*-test. The experiment was repeated independently three times.

Further, we characterized CD8^+^ TILs in B16-vec and B16-IL-36β tumors by flow cytometry. Interestingly, it was found p-S6 level of CD8^+^TILs was significantly upregulated by tumoral expression of IL-36β tumors compared with the control (Figure 6A). Then, we noted that the percentages of CD8^+^ TILs in CD45^+^ population were also increased in B16-IL-36β tumors compared with B16-vec tumors (Figure 6B). Taking into account that the numbers of CD45^+^ cells in per 10^5^ single cell suspension from B16-IL-36β tumors were more than 4-fold of that in B16-vec tumors (Figure S5A, left panel), the number of CD8^+^ TILs in B16-IL-36β tumors was greatly enhanced. Further, we examined the expression of Ki67 in CD8^+^ TILs by flow cytometry, which was significantly upregulated in CD8^+^ TILs from B16-IL-36β tumors (Figure 6C), suggesting that the increase of CD8^+^ TILs, in a large part, attributed to local IL-36β-mediated proliferation. The effective antitumor immune responses of CD8^+^ TILs can be characterized by the release of effector cytokines, such as IFN-γ. In order to reveal whether IL-36β promoted the antitumor function of CD8^+^ TILs, we examined IFN-γ expression in CD8^+^ TILs. The results showed that the percentages of IFN-γ^+^CD8^+^ TILs from B16-IL-36β tumors were evidently increased compared with B16-vec tumors (Figure 6D).

Additionally, the percentages of NK and γδT cells in CD45^+^ population in B16-IL-36β tumors were also augmented compared with B16-vec tumors, while the percentage of B cells was decreased (Figures S5B,C). Moreover, neutrophilic myeloid-derived suppressor cells (NMDSCs), defined by CD11b^+^Gr-1^hi^, were reduced, and MHC-II expression on NMDSCs and CD11b^+^Gr-1^int^ monocytic MDSCs (MMDSCs) was obviously upregulated in B16-IL-36β tumors (Figure S5D), which was basically consistent with the findings in B16-IL-36γ tumors (21).

Therefore, IL-36β could also promote mTORC1 activation antitumor function of CD8+ TILs in the tumor microenvironment, leading to a significant enhancement of antitumor immune responses.

## Discussion

CD8^+^ TILs in the tumor microenvironment are usually functionally exhausted (28, 29). Therefore, exploring effective means to recover CD8^+^ TIL antitumor function will contribute to tumor eradication. Clinically, blocking immune checkpoints by antibodies can restore CD8^+^ TIL function and has achieved a good therapeutic effect in the treatment of tumors. In addition, some cytokines can effectively stimulate CD8^+^ TIL function and have been approved for clinical treatment of tumors (30). Moreover, engineered T cells such as chimeric-antigen receptor T cells (CAR-T) is very powerful to eliminate tumors (31, 32). However, immunotherapy based on the blockade of immune checkpoints is effective only for a small portion of patients in that it relies on the amplification of spontaneous antitumor immune responses (33–35). At the same time, current CAR-T cell therapy often causes cytokine-releasing syndrome (CRS), among of which activation of NF-κB signal pathway play an important role. In contrast, cytokines are more effective in expanding tumor-specific CD8^+^ TILs with relatively small side effects.

IL-36 cytokines were initially found to effectively activate CD4^+^ T lymphocytes and DCs (19, 36). Recently, we found that IL-36γ could profoundly boost CD8^+^ T function, resulting in enhancement of antitumor immune responses (20, 21). In this study, it was further proved that IL-36β significantly promoted CD8^+^ T cell activation, even at an extremely early stage upon stimulation. This effect could be interpreted as that IL-36R was constitutively expressed on naïve CD8^+^ T cells and persistently maintained on activated CD8^+^ T cells (20, 21). Additionally, it was revealed that intratumoral IL-36α expression positively correlated with the number CD8^+^ TILs and its overexpression in hepatocellular carcinoma cell lines enhances CD8^+^ T cell infiltration into tumor (37). Therefore, IL-36 cytokines can be considered as appropriate agonists to boost antitumor function of CD8^+^ T cells.

However, the underlying mechanism of IL-36-promoted activation of CD8^+^ T cells remains exploration. In this study, our data substantiated that IL-36β obviously activated mTORC1 signal, while suppression of mTORC1 inhibited IL-36β-mediated CD8^+^ T cell activation, expansion and proliferation, suggesting that IL-36β-promoted CD8^+^ T cell activation was dependent on mTORC1. The effect of IL-36β/mTORC1 axis on CD8^+^ T cell activation could be explained by the following mechanisms. Metabolism and synthesis of biological molecules are necessary for T cell activation and proliferation (12, 38, 39). During this course, aerobic glycolysis is employed as an important pathway for biosynthesis (40, 41). By integrating multiple signals such as cytokine-derived stimulation, IL-36β-promoted mTORC1 activation has a crucial role in mediating the generation of highly glycolytic and potent effector CD8^+^ T cells (42, 43).

However, activation of mTORC1 in tumor cells was also reported to participate in promoting tumor progression and associated with poor prognosis in cancer (44–46). IL-1RAcP is expressed in a multitude of cancer cells and IL-36 signals through IL-36R/IL-1RAcP. Such facts indicate that when adopting IL-36 cytokines as immunotherapeutic materials, it should be carefully considered the expression levels of IL-36R as well as IL-1RAcP on tumor cells and whether IL-36/IL-36R/IL-1RAcP can significantly promote tumor growth. Interestingly, based on expression on chronic myeloid leukemia (CML), IL-1RAcP has been recently used as a tumor antigen target for CAR-T immunotherapy approach with no apparent effect on the hematopoietic system (47). This suggests that IL-36R/IL-1RAcP may be used as a tumor-associated antigen for CAR-T immunotherapy cell targeting.

Further, the signal pathways leading to IL-36β-mediated mTORC1 activation were explored. IL-36β in conjunction with TCR signal obviously increased phosphorylation of Akt, followed by enhancement of mTORC1 activation, while inhibiting PI3K suppressed IL-36β induced-mTORC1 activation. These data suggested that IL-36β-promoted mTORC1 activation was dependent on PI3K/Akt pathway. Simultaneously, inhibiting PI3K suppressed TCR signal-mediated S6 phosphorylation. Interestingly, the level of p-S6 in CD8^+^ T cells stimulated by IL-36β was downregulated to almost the same level as that of CD8^+^ T cells without IL-36β stimulation, indicating that PI3K/Akt pathway dominated IL-36β-triggered mTORC1 activation. Hence, PI3K/Akt pathway has a central role in IL-36β-promoted mTORC1 activation.

IKKs were also reported to exert an effect on activating mTORC1 (25, 26). Our study revealed that IL-36β, alone, could significantly enhance IKK activity, and had a synergistic effect with TCR signal, whereas inhibiting IKK partially downregulated IL-36β-mediated phosphorylation of S6, proving that IKK was also involved in IL-36β-triggered mTORC1 activation. It is well-known that NF-κB subunits have a key role in activating lymphocytes through moving into nucleus. IL-36 cytokines were previously described to activate the pathway leading to NF-κB in an IL-36R dependent manner (48). Hence, these findings validated a new mechanism that IL-36β could promote CD8^+^ T cell expansion and proliferation through IKK/mTORC1 pathway, in addition to that it can stimulate CD8^+^ T cell activation dependent on NF-κB pathway.

MyD88 is an important adaptor of IL-36/IL-36R signal (27). Our data proved that IL-36β-mediated mTORC1 activation of CD8^+^ T cells was almost abolished by MyD88 deficiency, resulting in arrest of expansion, proliferation, and cytokine production of CD8^+^ T cells, suggesting that MyD88 pathway was critical in IL-36β-promoted CD8^+^ T cell activation through activating mTORC1. These results were consistent with previous findings that MyD88 was necessary for the proliferation of Tc17 and Tc1 cells through the activation of mTOR (49).

Taken together, PI3K/Akt, IKK and MyD88 pathways were all involved in IL-36β-mediated mTORC1 activation.

Furthermore, we demonstrated that IL-36β exerted a similar effect on promoting mTORC1 activation and antitumor function of CD8^+^ TILs *in vivo*. Tumoral expression of IL-36β significantly inhibited tumor regression and prolonged the survival of tumor-bearing mice. At the same time, mTORC1 activation, proliferation and IFN-γ expression of CD8^+^ TILs, and CD8^+^ TIL number were all increased in tumor microenvironment. Hence, our data demonstrated that IL-36β could enhance CD8^+^ TIL antitumor function of through activating mTORC1. In addition, IL-36 cytokines could also indirectly function on CD8^+^ T cells through stimulating DCs and macrophages to induce type I-biased immune responses (18, 36). Therefore, IL-36 cytokines could promote CD8^+^ TIL antitumor function by multiple ways.

In summary, the current study demonstrated that IL-36β could promote CD8^+^ T cell activation and antitumor immune responses by activating mTORC1 through PI3K/Akt, IKK, and MyD88 pathways, and proved that IL-36β had potential application value in tumor immunotherapy.

## Materials and Methods

### Animals and Tumor Model

C57BL/6j (B6; H2Kb) mice (6–8 weeks old) were purchased from Shanghai Model Organism Center and housed in the specific pathogen-free facility of Medical School of Soochow University. All mice experiments were conducted under an institutional animal care and complied with committee-approved protocol. B16 cell-derived transfectants were injected intradermally (i.d.) to C57BL/6 mice, and the tumor size was monitored every 2 days.

### Plasmid Construction, Generation of IL-36β-Expressing Tumor Cell Lines, and Tumor Cell Culture

The pcDEF3-Dap10 vector was a gift from Dr. Lawrence Kane (University of Pittsburgh). The nucleotide sequence encoding mature peptide (S31-K183) of murine IL-36β was synthesized by Genewiz (Genewiz, New Jersey). In order to generate IL-36β-expressing tumor cell lines, firstly the nucleotide sequence encoding human CD8α signal sequence was fused to the 5′ end of IL-36β (S31-K183) sequence. Then the fused fragment was ligated into pcDEF3-Dap10 vector via BamH1 and Not1 to generate IL-36β expression construct, pcDEF3-Dap10-IL-36β. The inserted fragment was confirmed to be correct by DNA sequencing analysis. Further, the IL-36β expression vector was transfected into B16 using Lipofectamine 2000 (Invitrogen Life Technologies) according to the manufacturer's instructions. At the same time, empty vector (pcDEF3-Dap10) was transfected into B16 cells. After transfection for 24 h, cells were treated with G418 (Sigma) at a concentration of 600 mg/L and the survived cells after selection with G418 were further subcloned. Eventually, a high level of IL-36β expression, respectively, in the cell lines B16-IL-36β, but not in B16-vector (B16-vec) was confirmed by quantitative RT-PCR. B16, B16-vec, and B16-IL-36β cells were cultured in RPMI 1,640 plus 10% FCS.

### Purification and Stimulation of CD8^+^ T Cells

WT and MyD88^−/−^ mouse (a generous gift from R. Medzhitov, HHMI, Yale University, New Haven, CT, USA) spleens were picked and the red cells were lysed using ACK lysing buffer (Thermo Fisher Scientific). Then CD8^+^ T cells were purified by positive selection using immunomagnetic beads according to the manufacturer's protocol (CD8α [Ly-2] MicroBeads, Miltenyi) and the purity of CD8^+^ T cells was identified to reach 95% by FACS determination with PE-conjugated anti-CD8β antibody (eBioscience). Naïve CD8^+^ T cells were stimulated with or without plate-bound anti-CD3 mAb (10 μg/ml) or in combination with anti-CD28 mAb (5 μg/ml), in the presence or absence of IL-36β (100 ng/ml or a series of concentrations as indicated in dose-dependent experiment), human IL-2 (20 U/ml), IL-12 (10 ng/ml), and IL-36β in combination with either human IL-2 or IL-12 for various lengths of time. Effector CD8^+^ T cells were stimulated with IL-36β (100 μg/ml), soluble anti-CD3 mAb (1 μg/ml), IL-36β in combination with soluble anti-CD3 mAb, IL-12 (10 ng/ml), or IL-12 in combination with IL-36β.

### Examination of IKK Activity, p-Akt, and p-S6 in CD8^+^ T Cells by Western Blot

WT or MyD88^−/−^ mice-derived CD8^+^ T cells were stimulated with or without anti-CD3 and anti-CD28 mAbs in the presence or absence of IL-36β for various lengths of time and then were gathered and lysed in complete lysis buffer including protease and phosphatase inhibitors. Protein sample was electrophoresed and transferred to nitrocellulose filters. The filters were then blocked with 5% skimmed milk powder in PBS containing 0.1% Tween 20 (PBST) for 2 h at room temperature with gentle shaking. After blocking, the filters were incubated with 1: 1,000 diluted rabbit anti-mouse IκB, anti-phosphorylated Akt, anti-phosphorylated S6, or anti-tubulin antibody (Cell Signaling Technologies) in PBST containing 1% bovine serum albumin over night at 4°C. After washing with PBST for 5 times, filters were incubated with horseradish peroxidase-conjugated goat anti-rabbit IgG (Cell Signaling Technologies) at a dilution of 1:3,000 in PBST containing 1% skimmed milk for 1 h with gentle shaking. Filters were rinsed extensively in PBST and treated using chemiluminescence reagent (Cell Signaling Technologies). The immunoreactive bands were visualized using a Kodak Image Station. The density of protein bands was determined by densitometric analysis using the image software. For analyzing the role of PI3K/Akt and IKK pathways in IL-36β-induced mTORC1 activation, PI3K inhibitor (Wortmannin, Selleck) with a concentration of 20 nM and IKK inhibitor (IKK16, Selleck) with a concentration of 100 nM were adopted.

### Analysis of Phosphorylated S6 in CD8^+^ T Cells by Flow Cytometry

The phosphorylated ribosomal protein S6 intracellular staining was measured in activated CD8^+^ T cells as per manufacturer's protocol by flow cytometry. Briefly, cells were permeabilized for 20 min with fixation/permeabilization kit (eBioscience, San Diego, CA) and then stained with Alex Flour 488-conjugated anti-phosphorylated S6 (Cell Signaling Technologies). Data were acquired using a FACS flow cytometer (BD Biosciences, San Jose, CA) and analyzed by FlowJo software (TreeStar Inc., San Carlos, CA).

### Analysis of Tumor-Infiltrating Lymphocytes and Myeloid-Derived Suppressor Cells

Tumor masses were minced and digested with a mixture of 0.3 mg/ml DNase I (Sigma-Aldrich) and 0.25 mg/ml Liberase TL (Roche) in serum-free RPMI at 37°C for 25 min. Then the digested pieces were gently pressed between the frosted edges of two sterile glass slides, and the cell suspension was filtered through a 40 μm cell strainer (BD Biosciences). The immune subsets in the tumors were determined by flow cytometry and data were acquired using a FACS flow cytometer (BD Biosciences, San Jose, CA). For intracellular IFN-γ staining, harvested cells were stimulated with PMA (10 ng/ml) and ionomycin (1 μg/ml) for 4 h and incubated for the last 1 h with brefeldin A (10 μg/ml). Cells were subjected to intracellular cytokine analysis with anti-IFN-γ antibody. The antibodies used for FACS include APC-conjugated anti-CD45, Pacific blue-conjugated anti-CD8, FITC-conjugated anti-IFN-γ, Alex Flour 488-conjugated anti-p-S6, PerCP/Cy5.5-conjugated anti-CD4, PE-conjugated anti-NK1.1, FITC-conjugated anti-TCRγδ, PE-Cy7-conjugated anti-CD11b, FITC-conjugated anti-Gr-1, and PE-conjugated anti-MHC-II (eBioscience). Data were analyzed by FlowJo software (TreeStar Inc., San Carlos, CA).

### Statistics

Data were analyzed with GraphPad Prism 6.0 software (GraphPad, San Diego, CA), and summarized and presented as mean ± standard error of the mean (SEM). For comparisons of the collected data, two-tailed unpaired *t-*test or one-way ANOVA test were performed with *p* < 0.05 considered statistically significant. The error bar in each group in figures represents the mean ± SEM.

## Data Availability

The raw data supporting the conclusions of this manuscript will be made available by the authors, without undue reservation, to any qualified researcher.

## Ethics Statement

The experimental protocols in this study were approved by the Medical Ethics Committee of Soochow University.

## Author Contributions

XZhao, XC, XS, PT, and CC designed and performed experiments, analyzed data, and prepared the manuscript. QZ, ML, RX, XY, CF, XZhu, YZ, and ZS designed and performed experiments, and analyzed data. XZhang prepared and revised the manuscript, and interpreted data. BL and XW supervised the study, prepared the manuscript, designed experiments, and analyzed data.

### Conflict of Interest Statement

The authors declare that the research was conducted in the absence of any commercial or financial relationships that could be construed as a potential conflict of interest.
