# Supplementary material for: IL-36β Promotes CD8+ T Cell Activation and Antitumor Immune Responses by Activating mTORC1
Source: Front Immunol. 2019 Aug 7;10:1803. doi: 10.3389/fimmu.2019.01803 (PMC6692458; doi:10.3389/fimmu.2019.01803)
Supplement: Supplementary file 1 [file Presentation_1.pptx]

## Slide 1
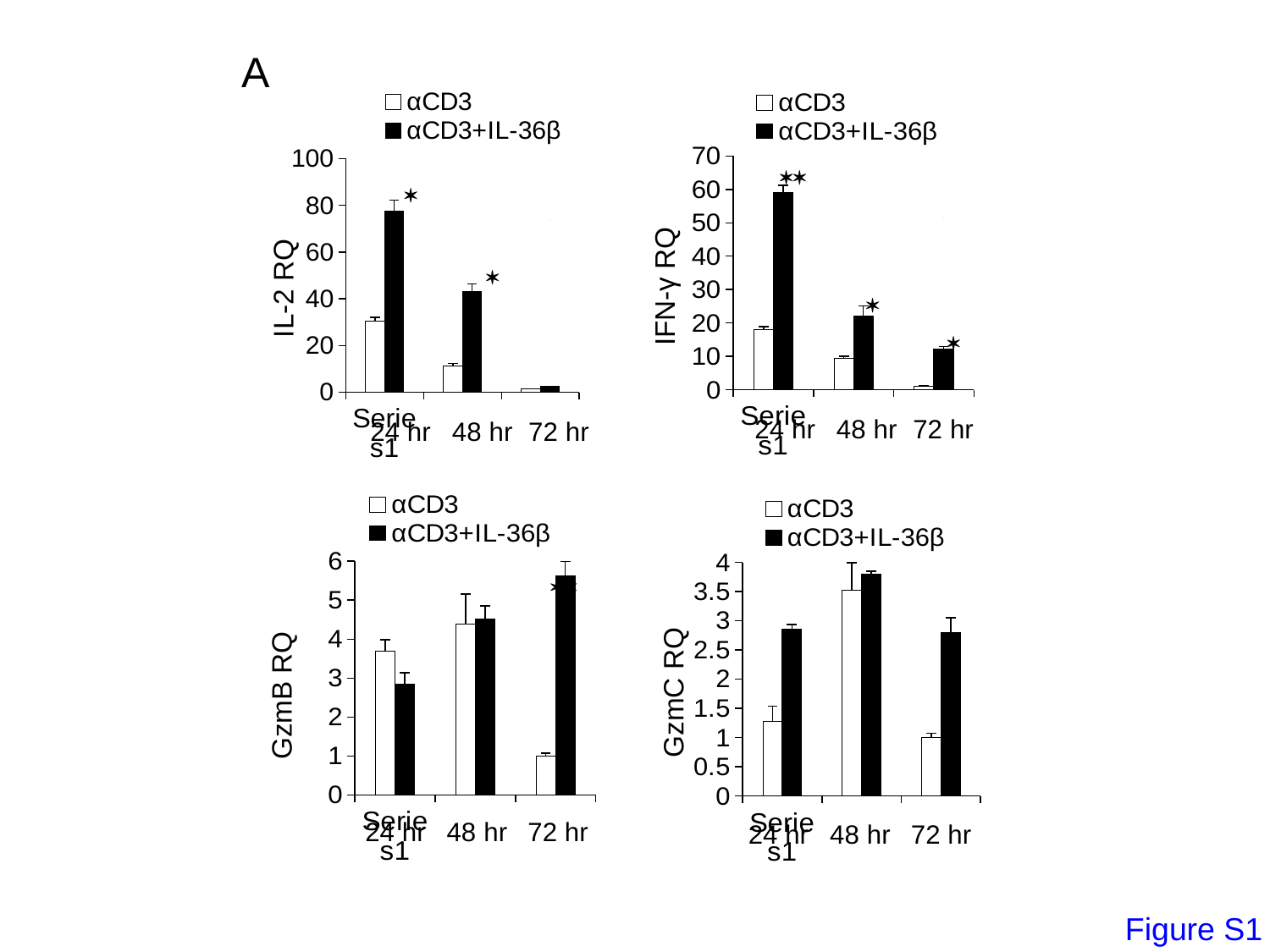

A
### Chart
| Category | αCD3 | αCD3+IL-36β |
|---|---|---|
| | 17.96762252481292 | 59.01011193988703 |
| | 9.321306659657845 | 22.16063470442768 |
| | 1.019171748894882 | 12.25887690869547 |
### Chart
| Category | αCD3 | αCD3+IL-36β |
|---|---|---|
| | 30.43542634255158 | 77.5331607791086 |
| | 11.1137906536271 | 43.0891600039372 |
| | 1.56870294775527 | 2.5139866004219 |


IFN-γ RQ
IL-2 RQ


24 hr
48 hr
72 hr
24 hr
48 hr
72 hr
### Chart
| Category | αCD3 | αCD3+IL-36β |
|---|---|---|
| | 3.68876889558248 | 2.84037932684203 |
| | 4.39267924191986 | 4.51371413227516 |
| | 1.00047866249188 | 5.622466270788735 |
### Chart
| Category | αCD3 | αCD3+IL-36β |
|---|---|---|
| | 1.272453188436641 | 2.858824641846038 |
| | 3.514677245409152 | 3.795337360456605 |
| | 1.006086723146836 | 2.794246429643596 |


GzmC RQ
GzmB RQ
24 hr
48 hr
72 hr
24 hr
48 hr
72 hr
Figure S1

## Slide 2
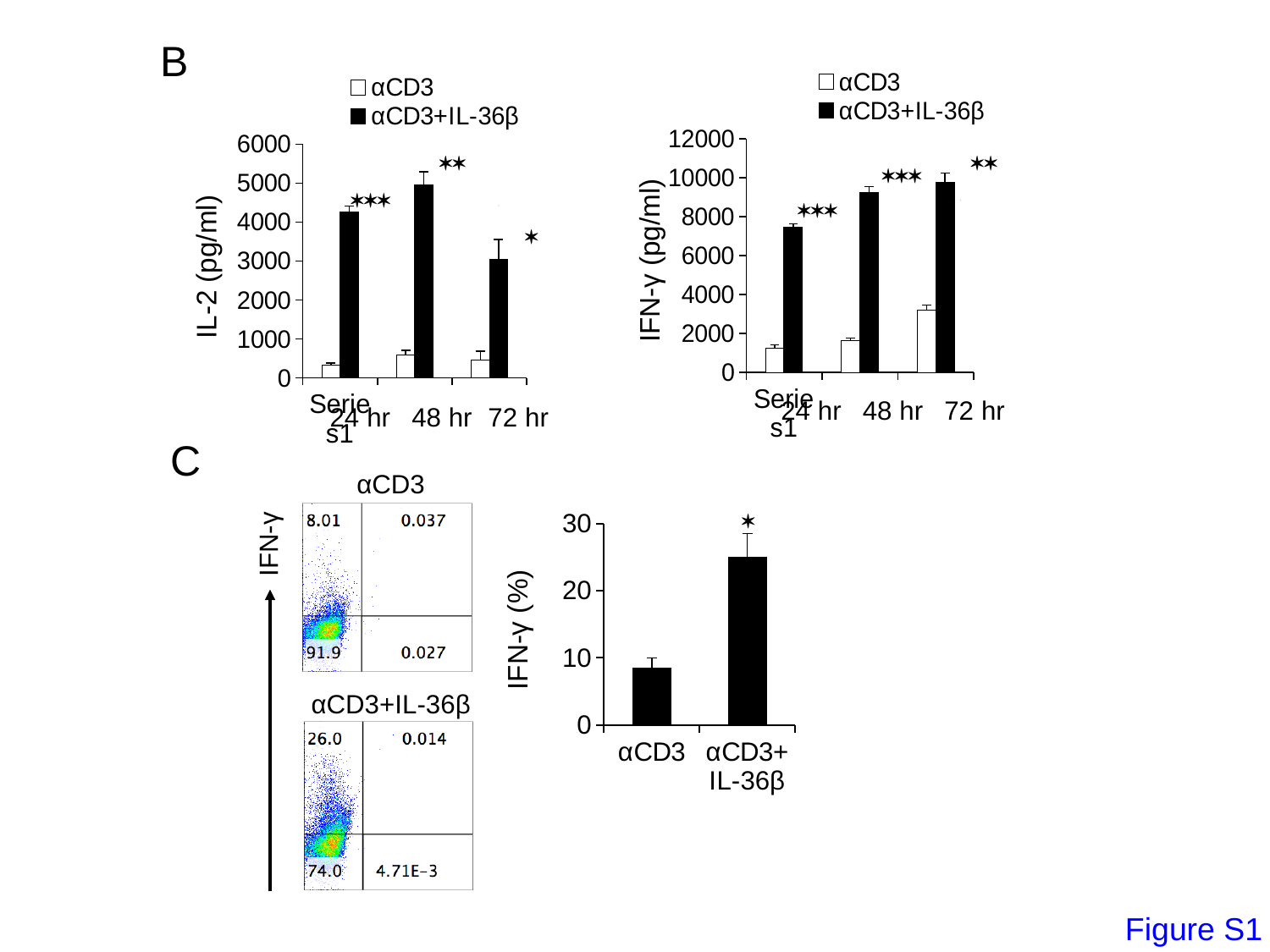

B
### Chart
| Category | αCD3 | αCD3+IL-36β |
|---|---|---|
| | 1255.0 | 7450.0 |
| | 1625.0 | 9250.0 |
| | 3200.0 | 9750.0 |
### Chart
| Category | αCD3 | αCD3+IL-36β |
|---|---|---|
| | 325.0 | 4250.0 |
| | 585.0 | 4950.0 |
| | 450.0 | 3050.0 |





IFN-γ (pg/ml)
IL-2 (pg/ml)
24 hr
48 hr
72 hr
24 hr
48 hr
72 hr
C
αCD3

### Chart
| Category | |
|---|---|
| αCD3 | 8.5 |
| αCD3+IL-36β | 25.0 |
IFN-γ
IFN-γ (%)
αCD3+IL-36β
Figure S1

## Slide 3
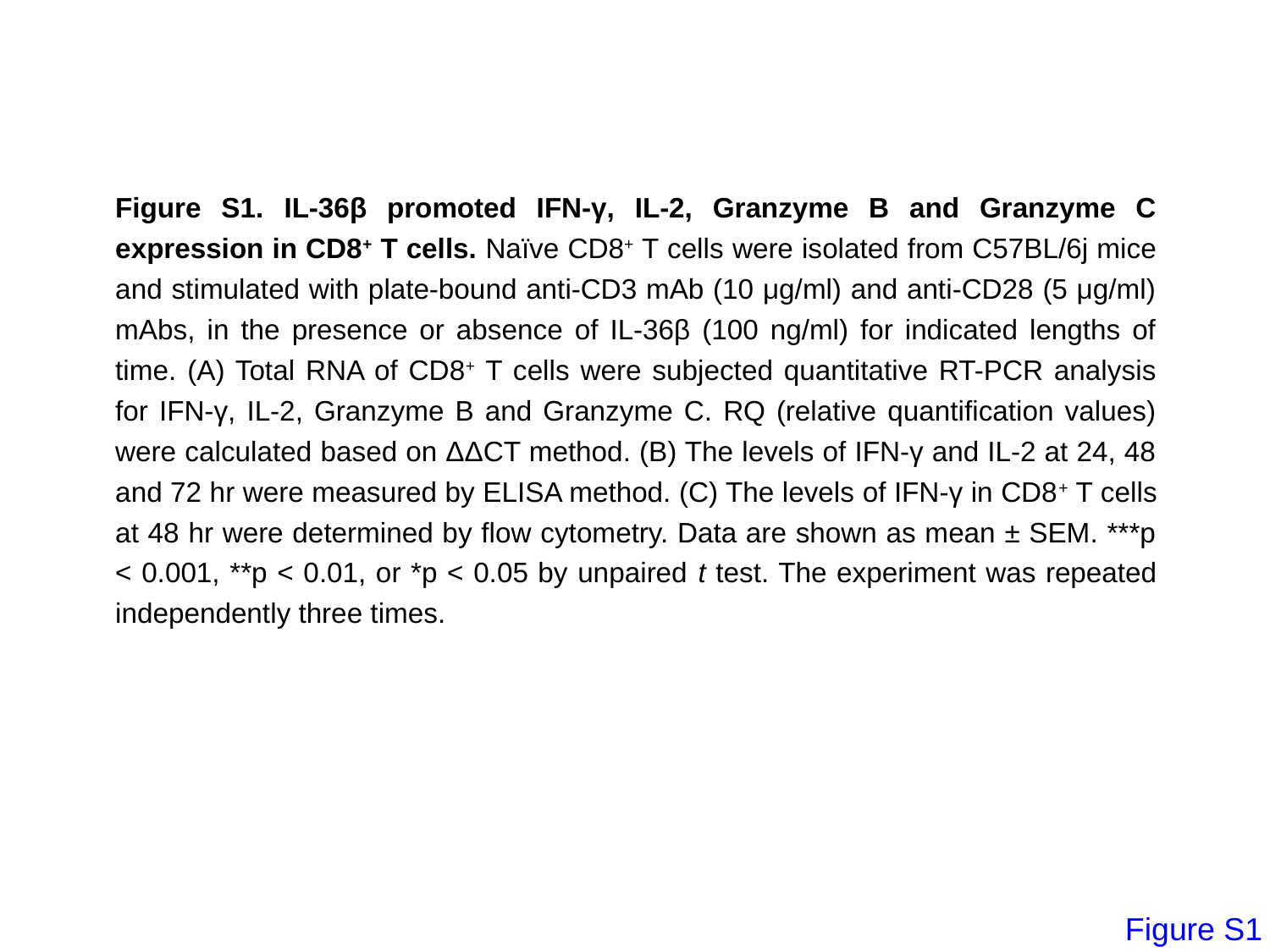

Figure S1. IL-36β promoted IFN-γ, IL-2, Granzyme B and Granzyme C expression in CD8+ T cells. Naïve CD8+ T cells were isolated from C57BL/6j mice and stimulated with plate-bound anti-CD3 mAb (10 μg/ml) and anti-CD28 (5 μg/ml) mAbs, in the presence or absence of IL-36β (100 ng/ml) for indicated lengths of time. (A) Total RNA of CD8+ T cells were subjected quantitative RT-PCR analysis for IFN-γ, IL-2, Granzyme B and Granzyme C. RQ (relative quantification values) were calculated based on ΔΔCT method. (B) The levels of IFN-γ and IL-2 at 24, 48 and 72 hr were measured by ELISA method. (C) The levels of IFN-γ in CD8+ T cells at 48 hr were determined by flow cytometry. Data are shown as mean ± SEM. ***p < 0.001, **p < 0.01, or *p < 0.05 by unpaired t test. The experiment was repeated independently three times.
Figure S1

## Slide 4
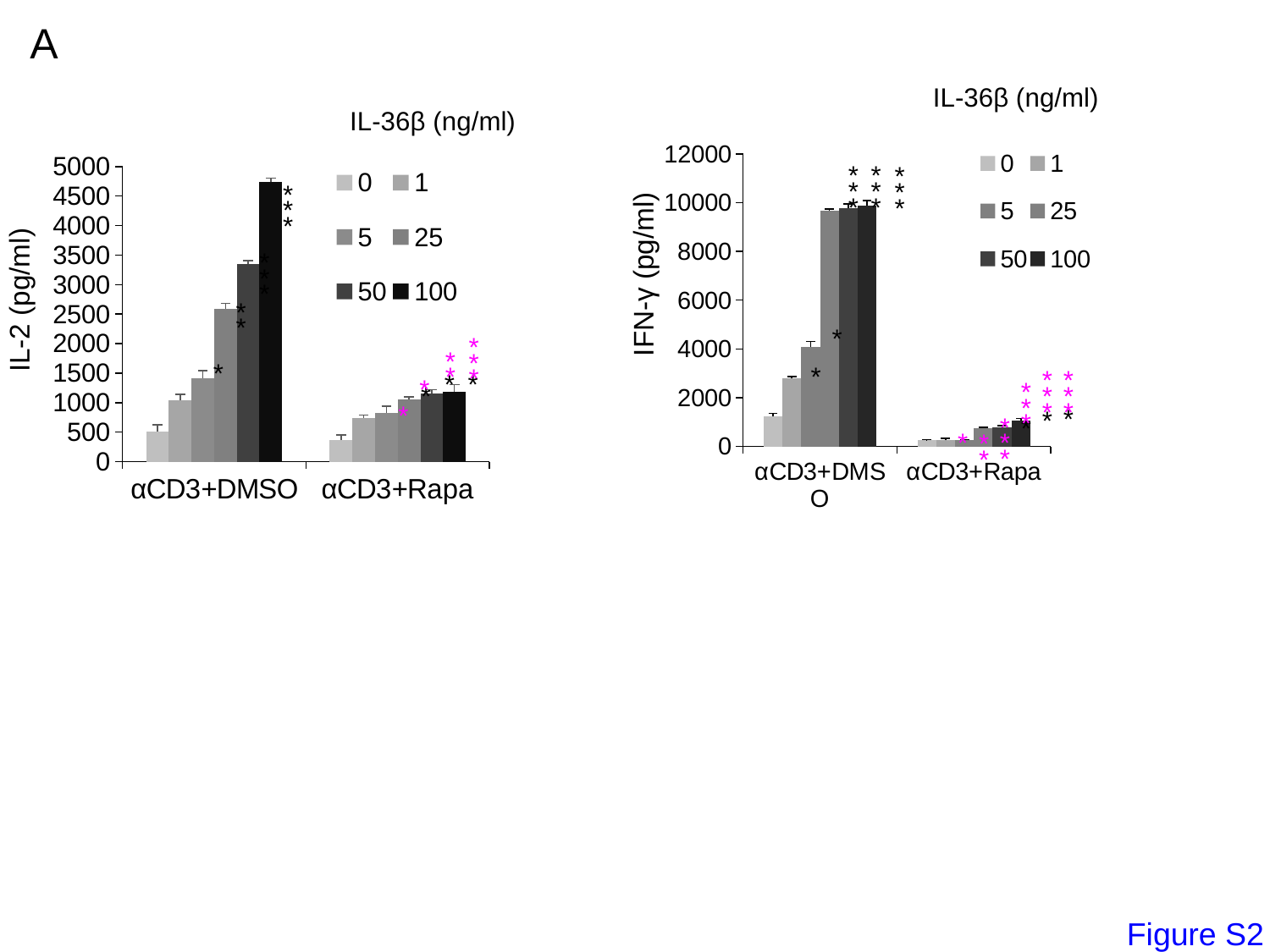

A
IL-36β (ng/ml)
IL-36β (ng/ml)
### Chart
| Category | 0 | 1 | 5 | 25 | 50 | 100 |
|---|---|---|---|---|---|---|
| αCD3+DMSO | 1248.955722 | 2807.017544 | 4076.858814 | 9657.477025999995 | 9786.967417 | 9878.863824999995 |
| αCD3+Rapa | 267.335004 | 263.1578947 | 258.980785 | 747.70259 | 797.8279029999997 | 1073.517126 |
### Chart
| Category | 0 | 1 | 5 | 25 | 50 | 100 |
|---|---|---|---|---|---|---|
| αCD3+DMSO | 513.920240666667 | 1033.10759966667 | 1416.85477833333 | 2590.66967633333 | 3350.63957866667 | 4735.13920233333 |
| αCD3+Rapa | 370.955605666667 | 739.653875 | 829.947328666667 | 1055.680963 | 1153.49887133333 | 1183.596689 | *
*
*
 *
*
*
 *
*
*
 *
*
*
 *
*
*
IFN-γ (pg/ml)
IL-2 (pg/ml)
 *
*
*
 *
*
*
 *
*
*
*
 *
*
*
 *
*
*
*
*
 *
 *
*
*
*
 *
*
*
*
 *
*
*
*
 *
*
Figure S2

## Slide 5
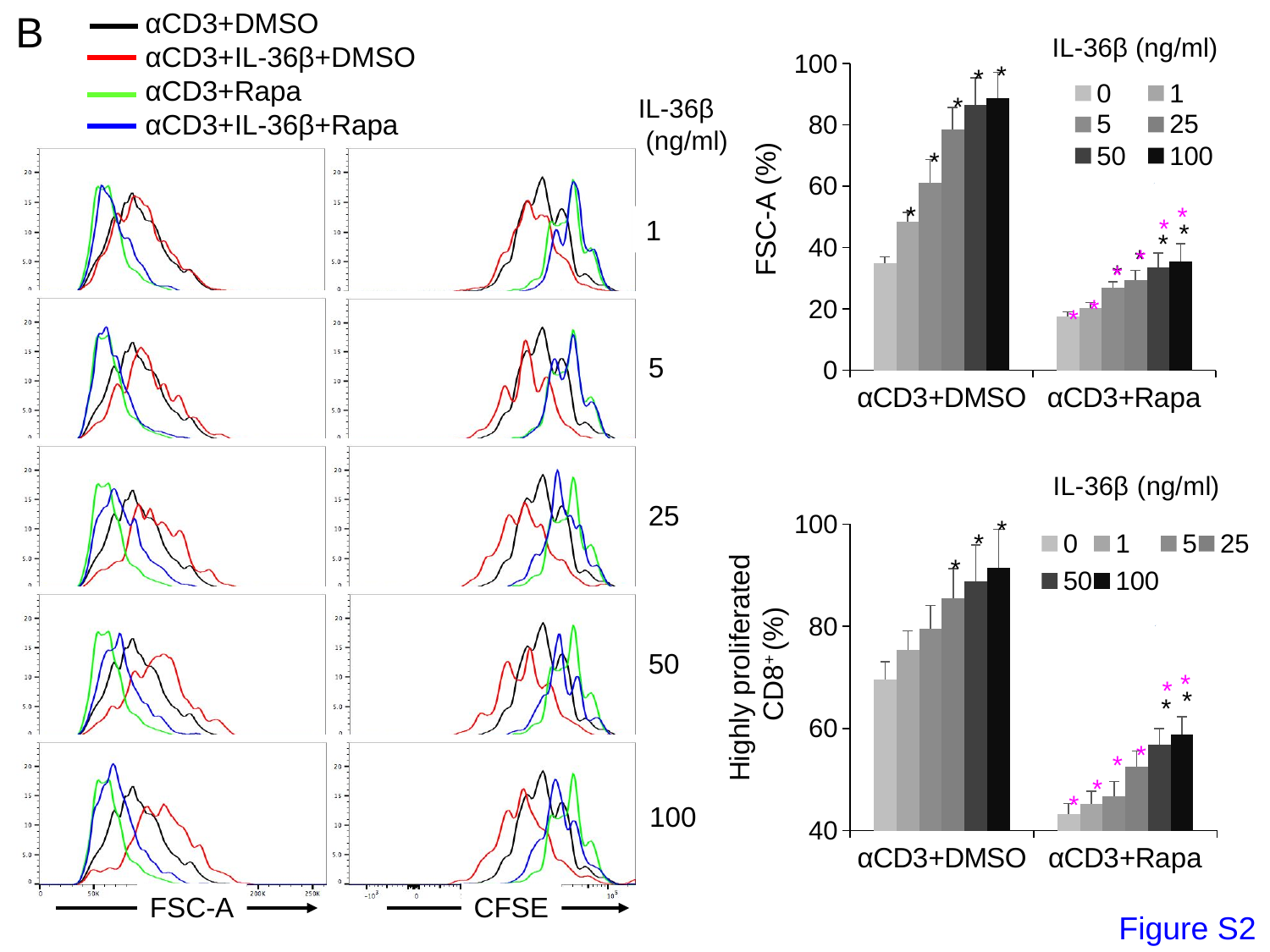

αCD3+DMSO
αCD3+IL-36β+DMSO
αCD3+Rapa
αCD3+IL-36β+Rapa
B
IL-36β (ng/ml)
### Chart
| Category | 0 | 1 | 5 | 25 | 50 | 100 |
|---|---|---|---|---|---|---|
| αCD3+DMSO | 34.8 | 48.5 | 61.2 | 78.5 | 86.5 | 88.6 |
| αCD3+Rapa | 17.5 | 20.2 | 26.8 | 29.3 | 33.6 | 35.5 | *
 *
 *
IL-36β
 (ng/ml)
 *
FSC-A (%)
 *
*
*
1
*
*
*
 *
*
 *
 *
 *
5
### Chart
| Category | 0 | 1 | 5 | 25 | 50 | 100 |
|---|---|---|---|---|---|---|
| αCD3+DMSO | 69.6 | 75.3 | 79.6 | 85.5 | 88.8 | 91.5 |
| αCD3+Rapa | 43.2 | 45.2 | 46.7 | 52.5 | 56.8 | 58.8 |25
 *
 *
 *
Highly proliferated
 CD8+ (%)
50
*
*
*
*
 *
*
 *
 *
100
FSC-A
CFSE
Figure S2

## Slide 6
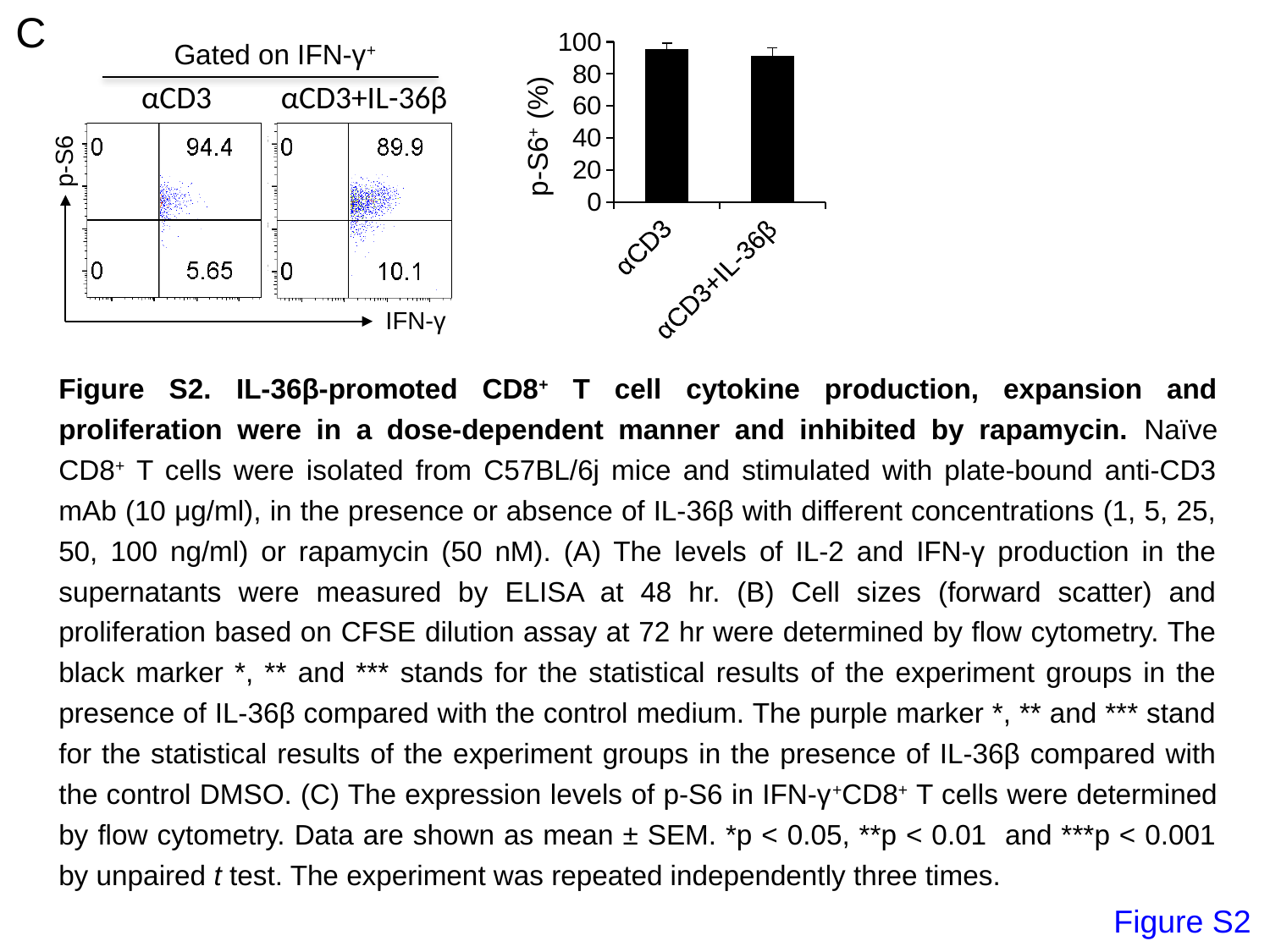

C
### Chart
| Category | |
|---|---|
| αCD3 | 95.0 |
| αCD3+IL-36β | 91.0 |Gated on IFN-γ+
αCD3
αCD3+IL-36β
p-S6+ (%)
p-S6
IFN-γ
Figure S2. IL-36β-promoted CD8+ T cell cytokine production, expansion and proliferation were in a dose-dependent manner and inhibited by rapamycin. Naïve CD8+ T cells were isolated from C57BL/6j mice and stimulated with plate-bound anti-CD3 mAb (10 μg/ml), in the presence or absence of IL-36β with different concentrations (1, 5, 25, 50, 100 ng/ml) or rapamycin (50 nM). (A) The levels of IL-2 and IFN-γ production in the supernatants were measured by ELISA at 48 hr. (B) Cell sizes (forward scatter) and proliferation based on CFSE dilution assay at 72 hr were determined by flow cytometry. The black marker *, ** and *** stands for the statistical results of the experiment groups in the presence of IL-36β compared with the control medium. The purple marker *, ** and *** stand for the statistical results of the experiment groups in the presence of IL-36β compared with the control DMSO. (C) The expression levels of p-S6 in IFN-γ+CD8+ T cells were determined by flow cytometry. Data are shown as mean ± SEM. *p < 0.05, **p < 0.01 and ***p < 0.001 by unpaired t test. The experiment was repeated independently three times.
Figure S2

## Slide 7
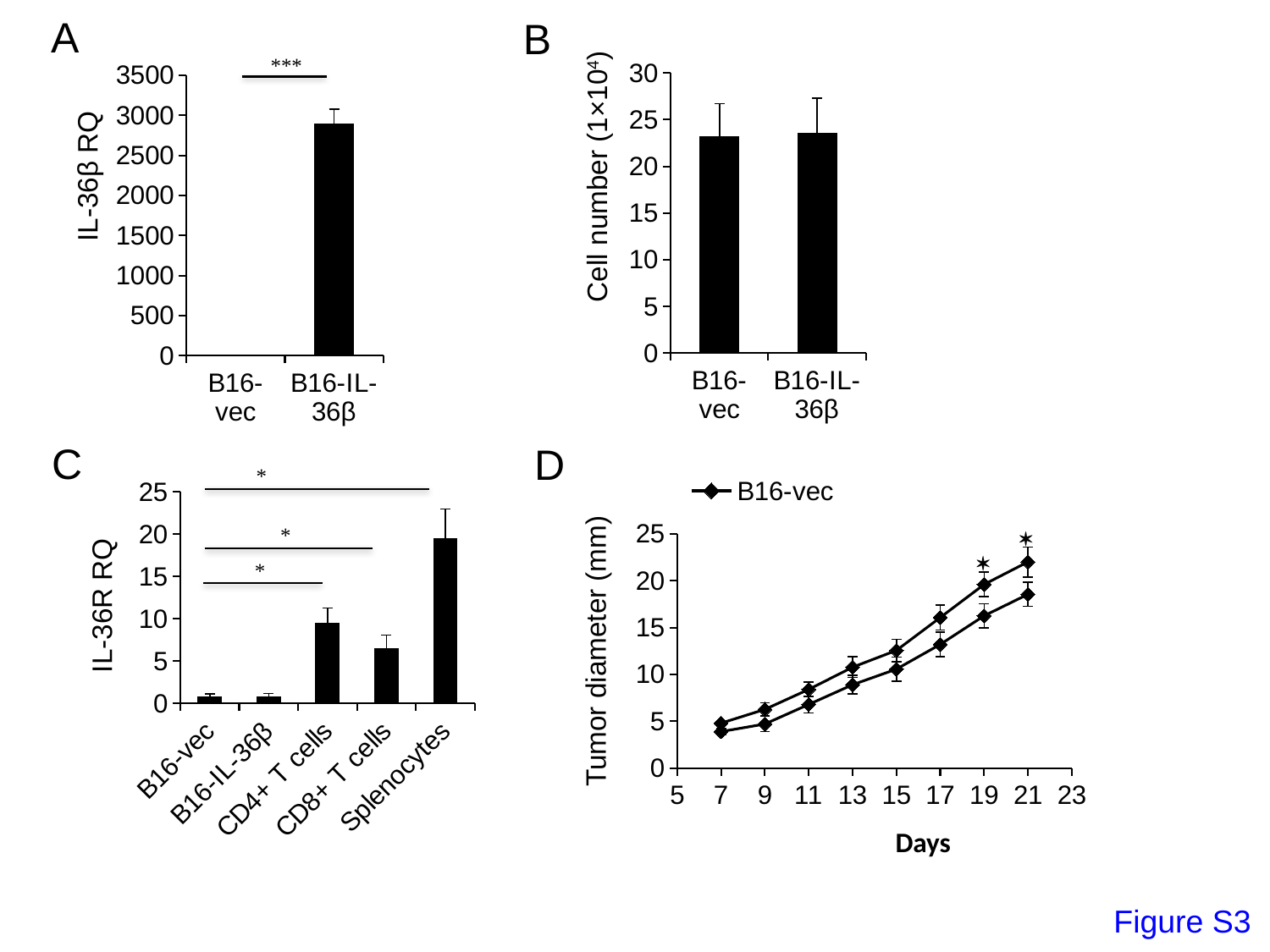

A
B
 ***
### Chart
| Category | |
|---|---|
| B16-vec | 23.2 |
| B16-IL-36β | 23.5 |
### Chart
| Category | |
|---|---|
| B16-vec | 1.003 |
| B16-IL-36β | 2887.1 |IL-36β RQ
Cell number (1×10^4^)
C
D
### Chart
| Category | B16-vec | B16-IL-36β |
|---|---|---| *
### Chart
| Category | |
|---|---|
| B16-vec | 0.75 |
| B16-IL-36β | 0.78 |
| CD4+ T cells | 9.5 |
| CD8+ T cells | 6.5 |
| Splenocytes | 19.4993 | 
 *
 
 *
IL-36R RQ
Tumor diameter (mm)
Days
Figure S3

## Slide 8
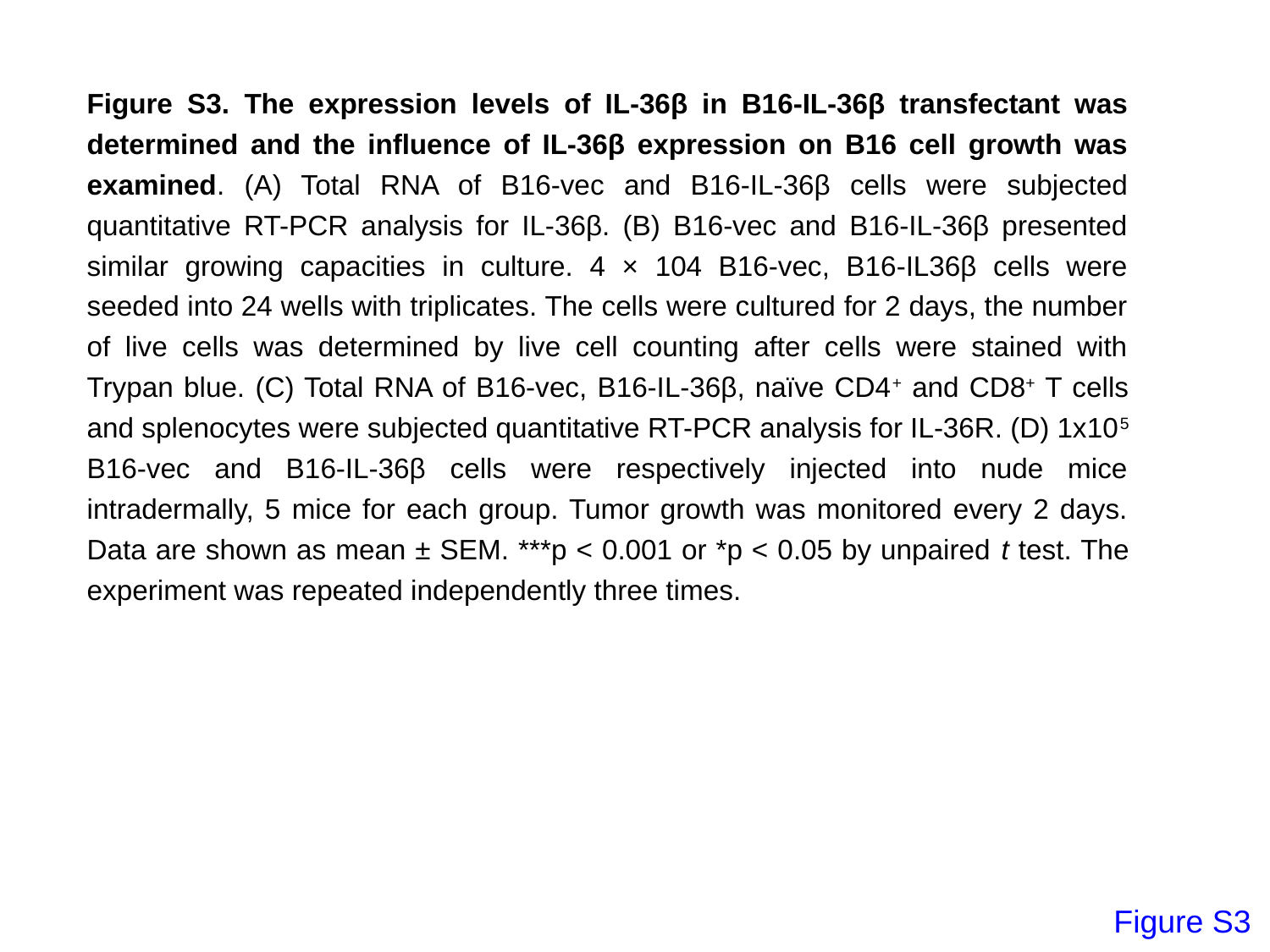

Figure S3. The expression levels of IL-36β in B16-IL-36β transfectant was determined and the influence of IL-36β expression on B16 cell growth was examined. (A) Total RNA of B16-vec and B16-IL-36β cells were subjected quantitative RT-PCR analysis for IL-36β. (B) B16-vec and B16-IL-36β presented similar growing capacities in culture. 4 × 10^4^ B16-vec, B16-IL36β cells were seeded into 24 wells with triplicates. The cells were cultured for 2 days, the number of live cells was determined by live cell counting after cells were stained with Trypan blue. (C) Total RNA of B16-vec, B16-IL-36β, naïve CD4+ and CD8+ T cells and splenocytes were subjected quantitative RT-PCR analysis for IL-36R. (D) 1x10^5^ B16-vec and B16-IL-36β cells were respectively injected into nude mice intradermally, 5 mice for each group. Tumor growth was monitored every 2 days. Data are shown as mean ± SEM. ***p < 0.001 or *p < 0.05 by unpaired t test. The experiment was repeated independently three times.
Figure S3

## Slide 9
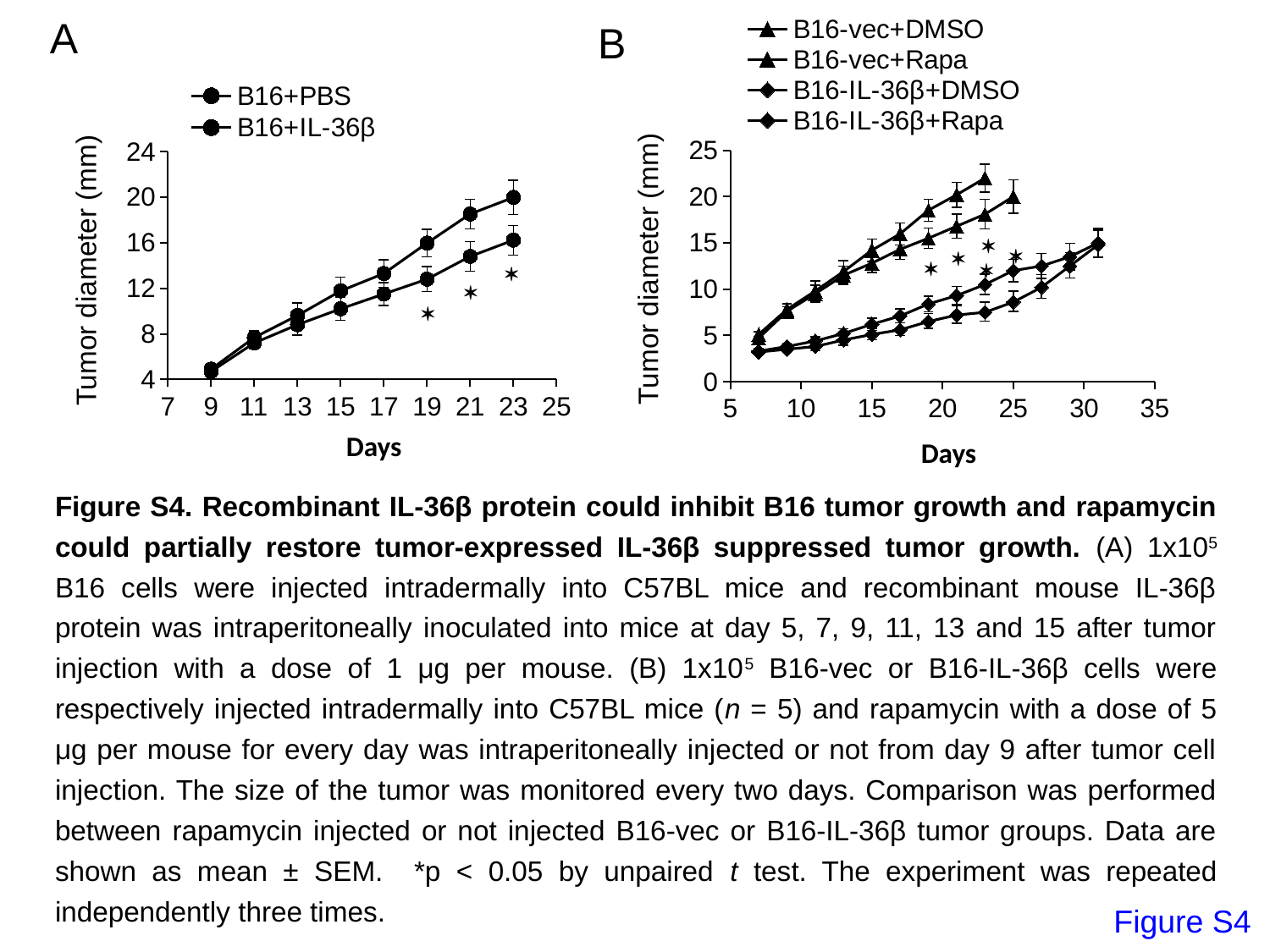

A
B
### Chart
| Category | B16-vec+DMSO | B16-vec+Rapa | B16-IL-36β+DMSO | B16-IL-36β+Rapa |
|---|---|---|---|---|
### Chart
| Category | B16+PBS | B16+IL-36β |
|---|---|---| 
 
 
 
 
Tumor diameter (mm)
Tumor diameter (mm)
 
 
 
Days
Days
Figure S4. Recombinant IL-36β protein could inhibit B16 tumor growth and rapamycin could partially restore tumor-expressed IL-36β suppressed tumor growth. (A) 1x10^5^ B16 cells were injected intradermally into C57BL mice and recombinant mouse IL-36β protein was intraperitoneally inoculated into mice at day 5, 7, 9, 11, 13 and 15 after tumor injection with a dose of 1 μg per mouse. (B) 1x10^5^ B16-vec or B16-IL-36β cells were respectively injected intradermally into C57BL mice (n = 5) and rapamycin with a dose of 5 μg per mouse for every day was intraperitoneally injected or not from day 9 after tumor cell injection. The size of the tumor was monitored every two days. Comparison was performed between rapamycin injected or not injected B16-vec or B16-IL-36β tumor groups. Data are shown as mean ± SEM. *p < 0.05 by unpaired t test. The experiment was repeated independently three times.
Figure S4

## Slide 10
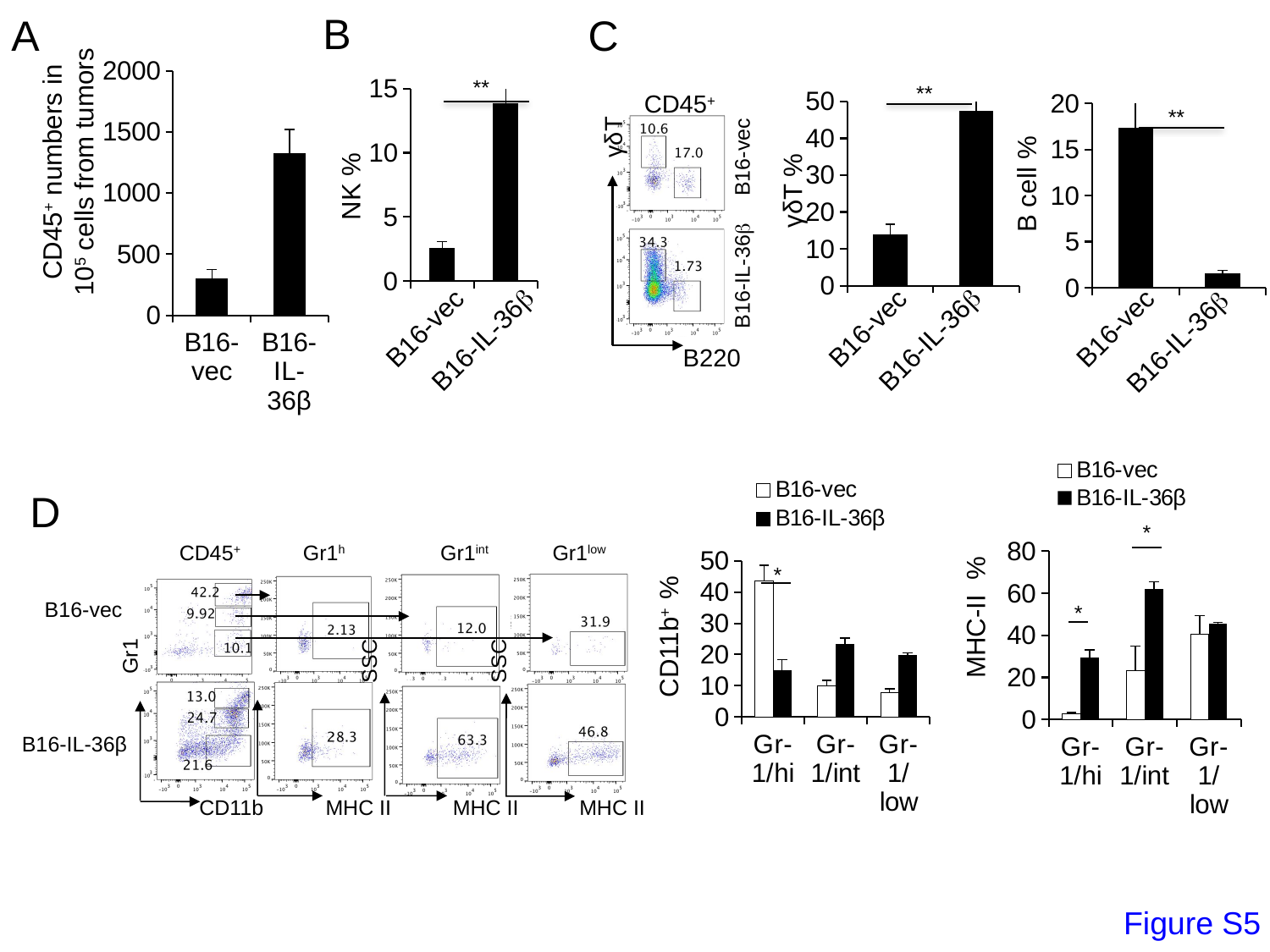

B
C
A
### Chart
| Category | |
|---|---|
| B16-vec | 300.0 |
| B16-IL-36β | 1320.0 | **
 **
### Chart
| Category | |
|---|---|
| B16-vec | 2.563 |
| B16-IL-36g | 13.86000000000002 |CD45+
### Chart
| Category | |
|---|---|
| B16-vec | 13.91 |
| B16-IL-36g | 47.4 |
### Chart
| Category | |
|---|---|
| B16-vec | 17.36 |
| B16-IL-36g | 1.59 | **
γδT
CD45+ numbers in
105 cells from tumors
B16-vec
B cell %
NK %
γδT %
B16-IL-36β
B16-vec
B16-vec
B16-vec
B16-IL-36β
B16-IL-36β
B16-IL-36β
B220
### Chart
| Category | B16-vec | B16-IL-36β |
|---|---|---|
| Gr-1/hi | 2.685 | 29.43 |
| Gr-1/int | 23.4 | 61.87 |
| Gr-1/low | 40.65 | 45.63 |
### Chart
| Category | B16-vec | B16-IL-36β |
|---|---|---|
| Gr-1/hi | 43.54 | 14.84 |
| Gr-1/int | 9.988000000000001 | 23.18 |
| Gr-1/low | 7.91 | 19.75 |D
 *
CD45+
Gr1h
Gr1int
Gr1low
 *
MHC-II %
B16-vec
 *
CD11b+ %
Gr1
SSC
SSC
B16-IL-36β
 CD11b
 MHC II
 MHC II
 MHC II
Figure S5

## Slide 11
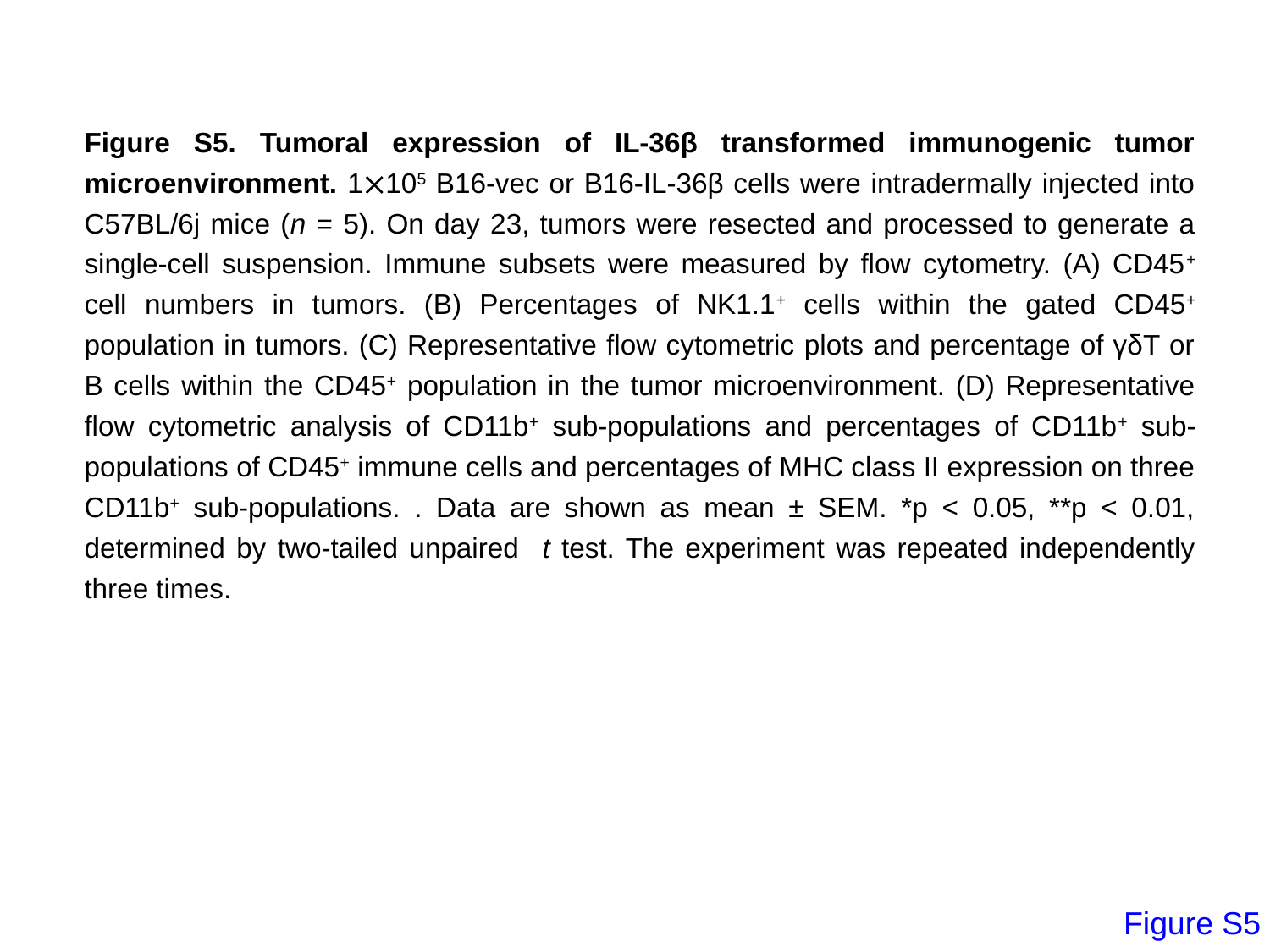

Figure S5. Tumoral expression of IL-36β transformed immunogenic tumor microenvironment. 1×10^5^ B16-vec or B16-IL-36β cells were intradermally injected into C57BL/6j mice (n = 5). On day 23, tumors were resected and processed to generate a single-cell suspension. Immune subsets were measured by flow cytometry. (A) CD45+ cell numbers in tumors. (B) Percentages of NK1.1+ cells within the gated CD45+ population in tumors. (C) Representative flow cytometric plots and percentage of γδT or B cells within the CD45+ population in the tumor microenvironment. (D) Representative flow cytometric analysis of CD11b+ sub-populations and percentages of CD11b+ sub-populations of CD45+ immune cells and percentages of MHC class II expression on three CD11b+ sub-populations. . Data are shown as mean ± SEM. *p < 0.05, **p < 0.01, determined by two-tailed unpaired t test. The experiment was repeated independently three times.
Figure S5
